# Peripheral Population Status and Habitat Suitability Assessment of the Kiang (*Equus kiang*) on the Eastern Tibetan Plateau

**DOI:** 10.3390/ani14192840

**Published:** 2024-10-02

**Authors:** Shuai Yang, Yi Yang, Bin Feng, Lu Hu, Xin Dong, Huiqin Dong, Wenke Bai

**Affiliations:** 1 Key Laboratory of Southwest China Wildlife Resources Conservation, China West Normal University, Nanchong 637009, China; yangs21711@126.com (S.Y.); yangyi3993@163.com (Y.Y.); fengbin9708@126.com (B.F.); huluxhsfdx@163.com (L.H.); gardenwdx@126.com (X.D.); dhuiqin0108@163.com (H.D.); 2 School of Ecology and Environment, Tibet University, Lhasa 850001, China; 3 Institute of Ecology, China West Normal University, Nanchong 637002, China

**Keywords:** kiang (*Equus kiang*), Tibetan Plateau, peripheral population, population size, habitat distribution

## Abstract

**Simple Summary:**

Shiqu County, in Sichuan Province, is the eastern edge of the distribution area of the kiang (*Equus kiang*) on the Tibetan Plateau. However, there is a lack of understanding about the kiang population and its habitat in this region. This study aims to assess the population and habitat status of kiangs. The results showed that the area of suitable habitat for kiangs is 3402.45 km^2^, with an estimated population of 1395.00 ± 272.20, and a population density of 0.41 ± 0.08/km^2^. These findings provide a scientific reference for the conservation of the kiang population and its habitat in the region.

**Abstract:**

Shiqu County, Sichuan Province, forms the eastern edge of the distribution area of the kiang (*Equus kiang*). It is important to assess the population and habitat status of peripheral populations, as these play a significant role in the development of conservation strategies for kiangs. Based on field transect data collected from July to August 2023, this study predicted the suitable habitat distribution of kiangs in Shiqu County using a MaxEnt model and estimated the population size and density using the ‘Distance’ package. Additionally, it analyzed the responses of the group size of kiangs to environmental factors in Shiqu County. The results showed that the area of suitable habitat for kiangs is 3402.45 km^2^, accounting for 13.51% of the total area of Shiqu County. The estimated population was 1395.00 ± 272.20, with a population density of 0.41 ± 0.08/km^2^. Additionally, the group size of kiangs was significantly positively correlated with the distance from the road and grazing site. The distance from the grazing site, elevation, and temperature annual range are the main environmental factors affecting the distribution of kiangs. This study initially revealed the peripheral population Status and suitable habitat of the kiang on the eastern Tibetan Plateau, and the potential threat of grazing interference and road obstruction. The research results can provide a scientific reference for the population and habitat protection of kiangs in this area.

## 1. Introduction

Peripheral populations are wildlife that inhabit the margins of a species’ distribution range and are often characterized by reduced genetic diversity or heightened environmental adaptability [1]. Peripheral populations of a species may hold particular value for the conservation of intraspecific biodiversity. However, due to their isolation and marginal nature, these populations often undergo inbreeding, leading to gene loss and genetic drift, which make them more vulnerable to the risk of extinction [2]. Additionally, the adaptation of peripheral populations to their specific habitats contributes to the preservation of population biodiversity and the expansion of their distribution range [3]. Peripheral populations experience greater pressures from ecological environmental changes, human activities, and climate change than core populations. Shiqu County, Sichuan Province, lies at the eastern edge of the distribution range of the kiang (*Equus kiang*) on the Tibetan Plateau. To protect and maintain the biodiversity of these peripheral populations, it is imperative to understand the current status of the kiang population and their habitats in this region.

Understanding the current status of species populations serves as an important foundation for wildlife conservation and management. Determining population size and aggregation patterns is fundamental to population status research [4]. Reliable estimates of wildlife population density and numbers are crucial for assessing survival status, monitoring population trends, and formulating effective conservation and management strategies [5]. The gregarious behavior of wildlife represents an adaptive response to natural environments. Group activities can mitigate the risk of individual predation and decrease the time spent on vigilance, thereby allowing individuals to allocate more time to behaviors that enhance their fitness [6]. Understanding the population size and aggregation characteristics of the kiang’s peripheral population is critical for comprehending its survival status, threat factors, and adaptive strategies.

Habitat constitutes the living environment upon which species rely, playing a pivotal role in the survival and reproduction of wildlife populations [7]. Habitat quality serves as a crucial indicator for assessing an ecosystem’s carrying capacity for species. It determines the stability of wildlife populations by influencing the suitability of their habitats [8]. Habitat assessment, from a macro-scale perspective, seeks to elucidate the distribution range and characteristics of species habitats and to analyze the impact of various ecological factors on the habitat suitability of the target species [9,10]. Peripheral populations typically exhibit a smaller distribution range or limited habitats, rendering them more sensitive to environmental changes. Therefore, conducting a habitat suitability assessment for the kiang is of paramount importance for the protection and restoration of this peripheral population’s habitat.

The kiang is a national first-class protected animal in China [11]. It primarily inhabits high-altitude regions and adjacent alpine grasslands at elevations ranging from 2700 to 5300 m [12]. Shiqu County, situated in the eastern part of the Tibetan Plateau, represents the sole distribution area of kiangs in Sichuan Province. It is the easternmost extent of the kiang’s distribution range. Currently, any research on kiangs across its range is relatively scant, and the status of the population and habitat in this region remains unclear [13]. With the continuous development of human society, anthropogenic activities such as grazing and road construction have resulted in the degradation of habitats for various wildlife species, including kiangs, in this region [14,15]. The habitat area for such species is progressively shrinking, accompanied by declining habitat quality and increasing fragmentation. To elucidate the survival status and potential threats to the peripheral population of kiangs in Shiqu County, this study analyzed the response patterns of the kiang population size to various environmental factors, calculated the population density of kiangs, and performed a habitat suitability assessment. The research findings can provide scientific reference for the protection of the kiang population and its habitat in this region, thereby promoting biodiversity conservation and ecological security within the alpine grassland ecosystem that is dominated by kiangs.

## 2. Materials and Methods

### 2.1. Study Area

Shiqu County (97°12′36″–100°41′24″ E, 31°43′48″–33°10′18″ N) is situated in the eastern part of the Tibetan Plateau, covering a total area of 25,191 km^2^. The topography slopes from northwest to southeast, with the northwestern part covering hilly plateaus and high plateaus, at elevations ranging from 3231 to 5334 m [16]. The study area features an average annual temperature of −0.9 °C and is rich in sunlight, with annual sunshine hours ranging from 2410 to 2530 h. The annual average precipitation is 569.6 mm. Natural grasslands cover 59.92% of Shiqu County’s total land area, making it the largest pastoral county in Sichuan Province, with the highest average elevation [17]. Yak grazing and husbandry constitute the primary means of production and livelihood in the region. The study area boasts rich biodiversity and serves as a habitat for many nationally protected wildlife species, including the kiang, snow leopard (*Panthera uncia*), white-lipped deer (*Cervus albirostris*), and several other rare wildlife species. Shiqu County is the easternmost extent of the kiang population’s distribution range.

### 2.2. Current Status of the Kiang Population

#### 2.2.1. Field Data Collection and Processing

**Figure 1:**
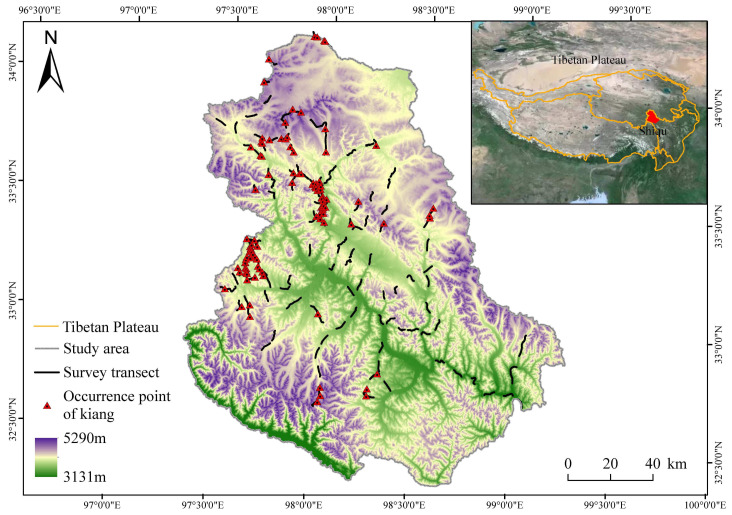
Survey transects and kiang occurrence points, study area.

From July to August 2023, a field survey was conducted in Shiqu County. Initially, stratified sampling was employed to establish transects, resulting in a total of 133 transects (each transect, measuring 3–5 km, was established along dirt roads, based on the surrounding environment and accessibility) covering alpine meadows, alpine shrubs, and alpine wetlands, which are the primary habitats of kiangs in the region (Figure 1) [18]. To avoid double counting, each transect was surveyed and completed within the span of a single day. Surveys were conducted along the established transects, and any sightings of animals or their traces (the feces of kiangs where there has been no recent grazing by herders in the vicinity) were recorded. The recorded data encompassed the number of individuals, sex, and age of the observed kiangs, as well as geographic coordinates, azimuth angle, distance from the kiang, and disturbance factors at the observation points. The actual distribution points of kiangs were determined by adjusting the geographic coordinates of the observation points based on the distance to the kiangs and the angle between the transect direction and the line of sight to the kiang. Additionally, the vertical distances between the actual distribution points and roads, water, and grazing points (of the yak groups herded by the herders that were observed during the survey) were calculated.

#### 2.2.2. Estimation of Kiang Population Size

The estimation of kiang population density was conducted utilizing the “Distance” package within the R4.2.1 software [18,19]. The corrected 106 group points of kiangs, the number of individuals in each group, and the vertical distances from the roads were imported into the software. By applying the detection function f(x), the probability of detecting kiang groups at a vertical distance x was calculated, resulting in the estimation of population density and the 95% confidence interval. The detection function comprises two components: the key function and the series expansion. The key functions encompass uniform distribution, half-normal distribution, and hazard-rate detection. The series expansions, employed to adjust the key functions, encompass cosine, simple polynomial, and Hermite polynomial. The Akaike information criterion (AIC) was utilized as the model evaluation standard. Different combinations of key functions and series expansions were compared based on their AIC values, with the model exhibiting the smallest AIC value being selected as the optimal model [18,20]. The population size of the kiangs was determined by integrating the estimated population density with the area of suitable habitat for the species.

#### 2.2.3. Analysis of Kiang Population Aggregation Characteristics

The analysis of the kiang population’s aggregation characteristics was performed utilizing R4.2.1 software. The number of individuals in each kiang group was regressed against the distances to roads, water sources, and grazing points, employing ordinary least squares regression (OLS). A significance level of 0.05 was established to analyze the response patterns of group size to various environmental factors.

### 2.3. Habitat Evaluation of Kiang

#### 2.3.1. Species Occurrence Points

The species occurrence data comprised 106 kiang group points and 107 trace points. To mitigate the spatial autocorrelation effect resulting from closely located occurrence points in the model analysis [21], duplicate occurrence points within a 1 km × 1 km area were removed using the “ENMTools” package within the R4.2.1 software [22,23]. Ultimately, 188 kiang occurrence points were retained for the construction of the MaxEnt model.

#### 2.3.2. Acquisition and Processing of Environmental Factors

In this study, five categories of environmental variables were selected to contribute to the habitat suitability prediction model for kiangs: climate, topography, vegetation, water sources, and human activity. The climate variables encompassed 19 bioclimatic factors obtained from the WorldClim database (http://www.worldclim.org, accessed on 12 September 2023), with a resolution of 30”. Topographic variables included elevation, slope, and aspect, sourced from the Geographic Spatial Data Cloud platform of the Computer Network Information Center, Chinese Academy of Sciences (http://www.gscloud.cn/, accessed on 12 September 2023). Vegetation variables comprised land use types and the normalized difference vegetation index (NDVI). Land use types were acquired from the study area’s Forestry and Grassland Bureau, and NDVI information was derived from MODIS images (https://modis.gsfc.nasa.gov, accessed on 12 September 2023). Water source variables were represented by the distance to water sources, generated as an Euclidean distance layer using the vector data of rivers and lakes in the study area within ArcGIS10.8 software. Human impact variables include the distances to roads, settlements, and grazing points. These were generated as Euclidean distance layers for each grid cell to the nearest human impact feature using the vector data of roads, settlements, and grazing points in ArcGIS10.8 software.

Employing the BILINEAR method for continuous variables and the NEAREST method for discrete variables, the 30 environmental variables were resampled to a uniform resolution of 30 m × 30 m in ArcGIS10.8. To avoid multicollinearity among the environmental variables, the Pearson correlation coefficients were calculated, and variables exhibiting Pearson coefficients greater than 0.7 were excluded [24].

#### 2.3.3. Model Optimization and Construction

The stability of the MaxEnt model is significantly influenced by two critical parameters: feature classes (FC) and the regularization multiplier (RM) [25,26]. Therefore, this study adopted the method of Cobos et al. [27] and employed the “ENMeval” package in R4.2.1 to optimize the MaxEnt model. The optimized model utilized the filtered species distribution points and environmental factor data. During the optimization process, the RM was set to start at 0.1, incrementing by 0.5 until reaching 6, resulting in 13 regularization multipliers. These were then combined with six feature types: “L”, “LQ”, “H”, “LQH”, “LQHP”, and “LQHPT”, resulting in a total of 78 combinations. Using the Akaike information criterion corrected for small sample sizes (AICc), the fit and complexity of various parameter combinations were evaluated to select the optimal model parameters for this species [28,29].

Based on the optimized model parameters, a species distribution model was developed using MaxEnt 3.4.1 software. To achieve this, 75% of the data was employed for model construction, while the remaining 25% was allocated for model validation. The bootstrap method was executed 10 times, with the random seed enabled. The response curves and Jackknife options were utilized to comprehensively evaluate the impact of environmental factors on species distribution.

The result comprised continuous raster data ranging from 0 to 1. The model results were evaluated using the area under the ROC curve (AUC) value, which spans from 0 to 1. A higher AUC value indicates the superior predictive performance of the model [30]. The output results from the MaxEnt model were imported into ArcGIS 10.8 for further analysis. Based on the 10th percentile training presence logistic threshold [31,32], the model results were categorized into unsuitable and suitable habitats. Subsequently, the area of suitable habitat for kiangs was calculated.

## 3. Results

### 3.1. Current Status of the Kiang Population

#### 3.1.1. Aggregation Characteristics of the Kiang Population

**Figure 2:**
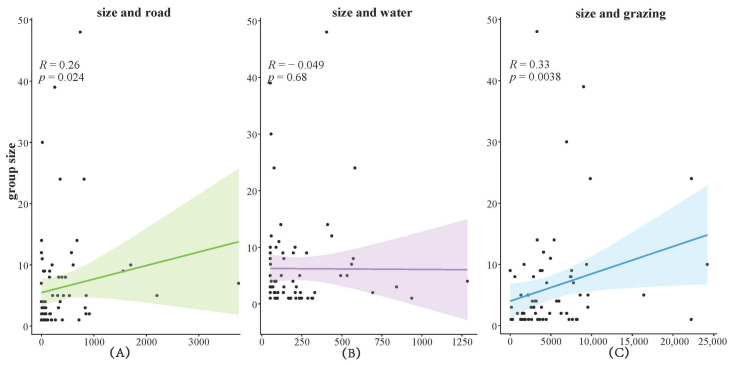
Regression analysis of kiang group size according to the distance from disturbance factors. Note: (**A**) shows the regression analysis of kiang groups size and distance from roads; (**B**) shows the regression analysis of kiang groups size and distance from water; (**C**) shows the regression analysis of kiang groups size and distance from grazing points.

During the transect surveys, a total of 106 kiang groups comprising 503 individuals were observed. The largest observed group contained 48 individuals, while the smallest consisted of a single individual. The group sizes of the kiangs exhibited a significant positive correlation with the distance from roads (*p* = 0.024) (Figure 2A) and the distance from grazing points (*p* = 0.0038) (Figure 2C).

#### 3.1.2. Kiang Population Size

**Figure 3:**
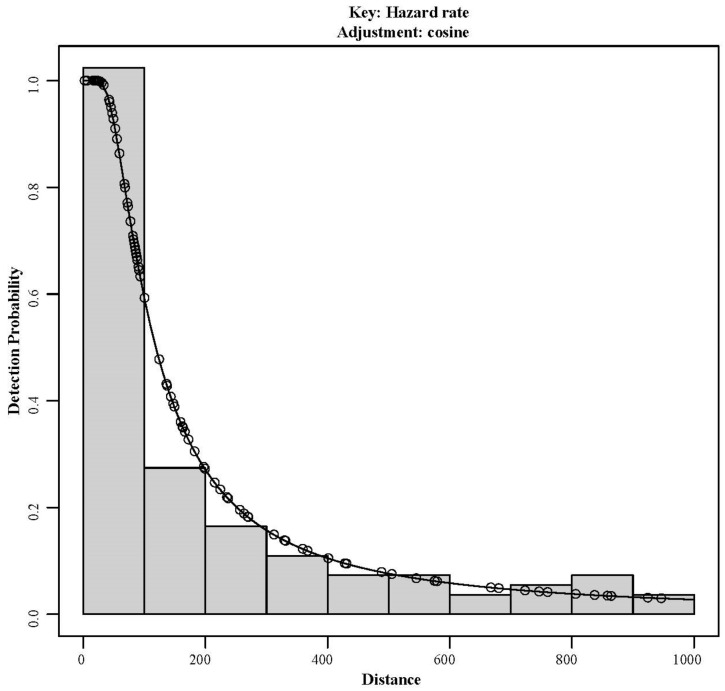
Detection function and histogram of vertical distances for kiang groups.

The optimal detection function combination for estimating the Kiang population was identified as the hazard-rate detection function, with a cosine series expansion (hazard-rate + cosine) (Table 1, Figure 3). The estimated density of kiangs in Shiqu County was 0.41 ± 0.08/km^2^ (95% CI: 0.25–0.57/km^2^). According to the MaxEnt model results, the suitable habitat area for kiangs in the study area was determined to be 3402.45 km^2^. Consequently, based on the estimated density and suitable habitat area, the estimated total population size of the kiangs was 1395.00 ± 272.20 (95% CI: 850.61–1939.40).

### 3.2. Habitat Suitability Evaluation for Kiangs

#### 3.2.1. Distribution of Suitable Habitats for Kiangs

**Figure 4:**
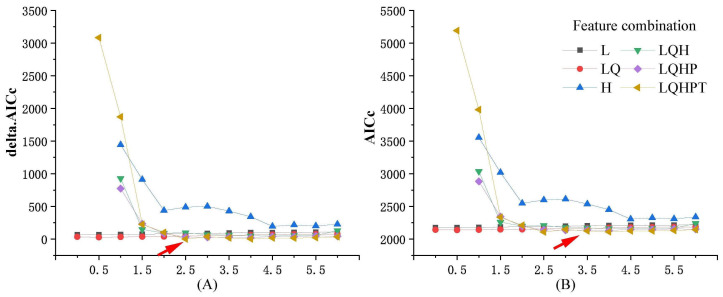
Performance of different parameter settings in the MaxEnt model for predicting kiang habitat suitability. (**A**) shows the different Feature combinations corrected delta.AICc; (**B**) shows the different Feature combinations corrected AICc. (The red arrow indicates the optimized model parameters with the lowest AIC value.)

The performance of the MaxEnt model with various parameter settings in predicting habitat suitability for kiangs is illustrated in Figure 4. The combination of “LQHPT” features with a regularization multiplier (RM) of 2.5 resulted in a deltaAICc of 0, indicating this as the optimal model (Figure 4A). The mean AUC value obtained from 10 model iterations was 0.897, demonstrating that the MaxEnt model possessed good predictive performance and high accuracy in forecasting a suitable habitat for kiangs.

**Figure 5:**
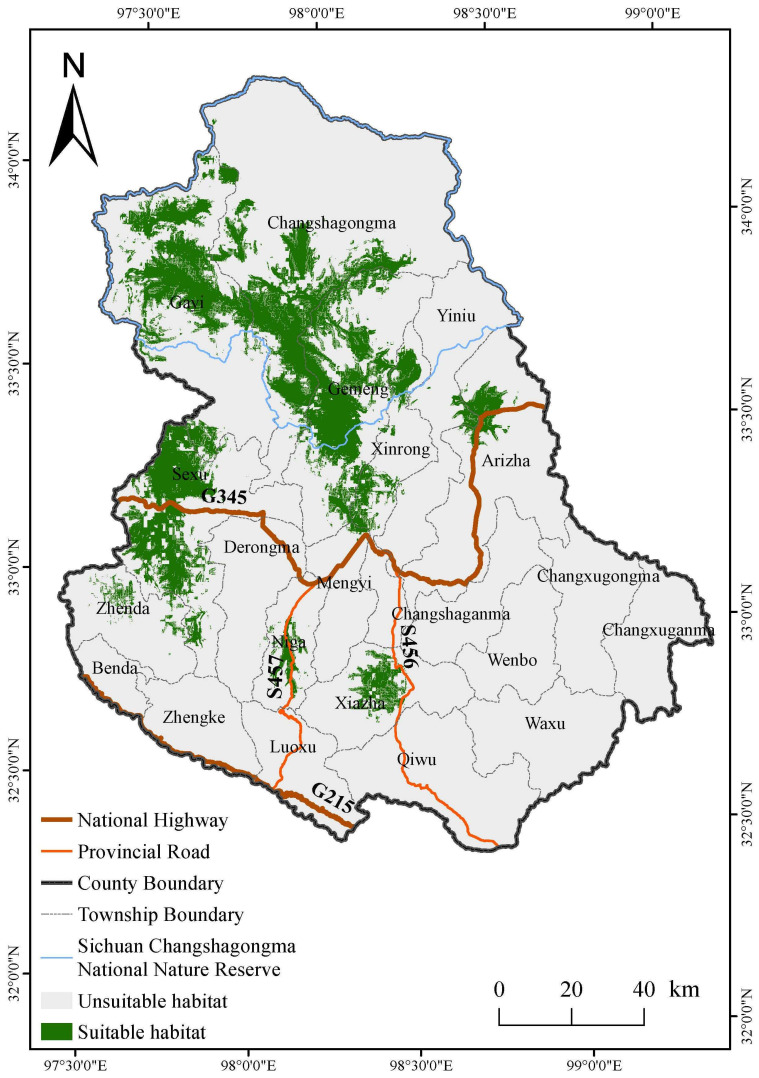
Suitable kiang habitat distribution.

The suitable habitat area for kiangs in the study area spans 3402.45 km^2^, constituting 13.51% of the Shiqu county’s total area. Spatially, the suitable habitat for kiangs is predominantly concentrated in and around six townships, covering approximately Changshagongma, Gayi, Gemeng, Sexu, Xiazha, and Arizha Town. The distribution is patchy, with larger contiguous patches of suitable habitat located around the Sichuan Changshagongma National Nature Reserve and the area surrounding Sexu Town (Figure 5).

#### 3.2.2. Factors Influencing Habitat Suitability

**Figure 6:**
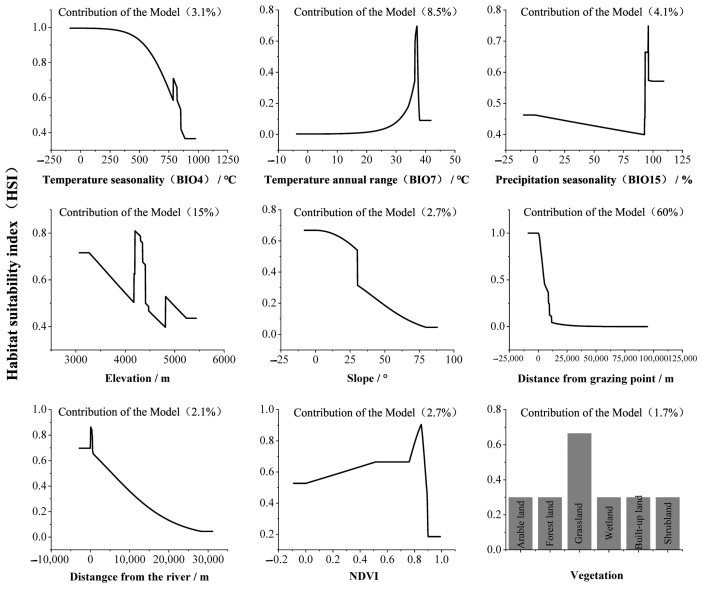
Response curves of kiang habitat suitability to dominant environmental factors.

The response curves of habitat suitability for kiangs with respect to various environmental factors and the contribution of each factor to the model are illustrated in Figure 6. The environmental factors contributing most significantly to habitat suitability for kiangs are the distance from grazing points, elevation, and temperature annual range (BIO7), with a cumulative contribution rate of 83.5%. Overall, the suitable habitat for kiangs is predominantly distributed within 0–5 km from grazing points, in gently sloping areas at an elevation of approximately 4000 m, and in grassland habitats with higher vegetation cover.

## 4. Discussion

Accurately estimating wildlife population sizes has been a long-standing objective for ecologists. In open and flat terrains like alpine meadows, transect methods are deemed reliable for surveying large ungulates such as the kiang [33,34]. This study estimated the density of kiangs in Shiqu County to be 0.41 ± 0.08/km^2^, which is lower than the estimated population density of 1.18 ± 0.34/km^2^ reported by Huangqingdongzhi et al. (2022) for the source region of the Yellow River source zone [18]. The differences in density estimates may be due to the fact that Shiqu County, compared to the Yellow River source zone, is located at the periphery of the kiang’s distribution range on the Tibetan Plateau [35], which leads to a significantly lower density of kiangs in that area. Additionally, surveys from different seasons can also lead to variations in the estimated population numbers of the species [33]. Different estimation methods can affect the results. To avoid interference from the estimation method, this study adopted the distance sampling method, using the “Distance” package within the R4.2.1 software to estimate the kiang population density [18,36].

In this study, the group size of kiangs exhibited a positive correlation with the distance from roads and grazing points. This pattern reflects the kiang’s aversion to road and grazing disturbances. The significant correlation between group size and the distance from roads and grazing points indicates that these human disturbances markedly influence the kiang’s grouping behavior and habitat selection [15,37,38]. The non-significant response of kiang group sizes to the distance from water sources may be attributed to the environment, which primarily consists of alpine meadows and wetlands. This terrain features numerous small, dispersed water bodies, thereby reducing the kiang’s dependence on larger water sources such as rivers.

Habitat fragmentation poses a significant challenge in the conservation of numerous wildlife populations [39]. In this study, the suitable habitats for kiangs in Shiqu County also exhibited considerable patchiness and fragmentation. The suitable habitats for kiangs in the study area are predominantly concentrated in four areas: the Sichuan Changshagongma National Nature Reserve and the vicinities of Sexu, Arizha, and Xiazha Town. The fragmentation of their habitat may be attributed to barriers created by major paved roads, including national and provincial highways. High traffic volumes impede the kiang’s ability to traverse these areas [40]. It was also observed that the suitable habitats for kiangs tend to avoid areas with high concentrations of grazing points. In summer, large numbers of herders engage in grazing activities that invade and degrade the suitable habitat areas for kiangs. Consequently, the kiangs in the study area face significant habitat fragmentation, primarily due to barriers created by major paved roads, such as national and provincial highways, and the impact of yak activities during summer grazing. Kiangs were not observed in some suitable habitats, which may be due to their mobility, resulting in their absence from these areas during the survey period. The response curve results for the habitat suitability of kiangs in relation to the distance from grazing points indicate that their suitable habitat is within 0–5 km of grazing points. This may be attributed to the widespread summer grazing in Shiqu County, where the occurrence points of kiangs are primarily concentrated within 5 km of grazing points. The actual avoidance behavior of the kiang primarily occurs within 200 m of grazing points, which influenced the model’s output.

This study revealed that the kiang population density in Shiqu County is relatively lower compared to the Yellow River source zone, while the degree of habitat fragmentation is notably high. The primary suitable habitats are located in the northern part of the study area, proximate to the Sanjiangyuan area. However, this region currently faces severe grazing disturbances, which are detrimental to the maintenance of the kiang population and the conservation of its habitat. To protect the habitat of the kiang population, it is recommended to formulate and implement relevant grazing management policies to mitigate the impact of grazing on their habitat [15,41]. Major roads, including national highways, also significantly impede the movement of kiangs between habitat patches. It is recommended to design and establish ecological corridors along the national and provincial highways in Sexu Town, Niga Town, and Arizha Town to ensure gene flow between fragmented populations and safeguard the genetic diversity of the kiang population in this region [38,42]. The current protected area planning exhibits certain deficiencies in safeguarding the kiangs’ habitat. It is advisable to consider incorporating parts of Sexu Town, where the kiang population density is relatively high, into the existing protected areas or establishing new protected zones.

## 5. Conclusions

The results of this study showed that the population density of the peripheral kiang population on the Eastern Tibetan Plateau is 0.41 ± 0.08/km^2^, with an estimated population of 1395.00 ± 272.20. This is consistent with the lower population densities typically observed in peripheral populations. The area of suitable habitat for the peripheral kiang population is 3402.45 km^2^, which constitutes 13.51% of the total area of Shiqu County. However, the distribution of suitable habitat is fragmented. The size of the kiang population is significantly positively correlated with the distance from roads and grazing points. The main environmental factors affecting kiang distribution are the distance from grazing points, altitude, and annual temperature range.

The findings of this study provide important scientific evidence for kiang conservation and will aid in developing more effective strategies. Future efforts should focus on monitoring the kiang population and its habitat, analyzing habitat fragmentation, studying the impact of roads, and exploring the feasibility of ecological corridors to enhance habitat connectivity.

## Figures and Tables

**Table 1:** Detection function model combination.

Model Type		AIC	ΔAIC
Key	Adjustment		
Hazard rate	Cosine	1321.202	0
Hazard rate	Hermite Polynomial	1322.111	0.909
Hazard rate	Simple Polynomial	1322.111	0.909
Half-Normal	Cosine	1326.834	5.632
Uniform	Cosine	1329.236	8.034
Half-Normal	Simple Polynomial	1361.466	40.264
Uniform	Simple Polynomial	1365.620	44.418
Half-Normal	Hermite Polynomial	1366.942	45.740
Uniform	Hermite Polynomial	1381.432	60.230

## Data Availability

The original contributions presented in the study are included in the Supplementary Materials, further inquiries can be directed to the corresponding authors.

## Supplementary Materials

The following supporting information can be downloaded at: https://www.mdpi.com/article/10.3390/ani14192840/s1.
