# Supplementary material for: Peripheral Population Status and Habitat Suitability Assessment of the Kiang (Equus kiang) on the Eastern Tibetan Plateau
Source: Animals (Basel). 2024 Oct 2;14(19):2840. doi: 10.3390/ani14192840 (PMC11475606; doi:10.3390/ani14192840)
Supplement: Supplementary file 1 [file animals-14-02840-s001.zip › animals-3224388- supplementary - aggie updated/data of kiang.pdf]

species	size	distance
kiang	1	90
kiang	14	25
kiang	9	68
kiang	1	16
kiang	5	864
kiang	1	42
kiang	39	256
kiang	3	17
kiang	2	20
kiang	1	91
kiang	1	149
kiang	3	329
kiang	1	162
kiang	4	33
kiang	12	6
kiang	30	27
kiang	5	545
kiang	48	746
kiang	11	22
kiang	4	359
kiang	1	30
kiang	9	159
kiang	1	579
kiang	5	312
kiang	4	52
kiang	1	197

kiang	2	81
kiang	1	806
kiang	1	84
kiang	1	137
kiang	10	84
kiang	2	87
kiang	1	143
kiang	1	269
kiang	1	100
kiang	1	86
kiang	1	124
kiang	1	136
kiang	2	166
kiang	2	172
kiang	2	72
kiang	7	87
kiang	3	85
kiang	1	199
kiang	1	82
kiang	24	1259
kiang	1	30
kiang	1	85
kiang	1	25
kiang	1	42
kiang	1	87
kiang	1	505
kiang	1	330

kiang	1	2
kiang	3	67
kiang	1	215
kiang	1	401
kiang	5	215
kiang	6	680
kiang	9	182
kiang	3	837
kiang	1	55
kiang	3	235
kiang	1	263
kiang	1	237
kiang	10	760
kiang	1	90
kiang	1	84
kiang	1	84
kiang	1	147
kiang	9	43
kiang	4	25
kiang	1	45
kiang	5	93
kiang	1	88
kiang	2	431
kiang	8	331
kiang	1	17
kiang	1	17
kiang	1	73

kiang	14	946
kiang	8	83
kiang	3	49
kiang	12	575
kiang	1	136
kiang	4	2
kiang	3	23
kiang	1	55
kiang	1	59
kiang	1	18
kiang	7	488
kiang	2	858
kiang	8	43
kiang	10	224
kiang	1	723
kiang	24	367
kiang	1	667
kiang	1	77
kiang	9	47
kiang	4	83
kiang	1	270
kiang	1	91
kiang	5	428
kiang	2	924
kiang	1	2
kiang	8	163
