# Supplementary material for: Peripheral Population Status and Habitat Suitability Assessment of the Kiang (Equus kiang) on the Eastern Tibetan Plateau
Source: Animals (Basel). 2024 Oct 2;14(19):2840. doi: 10.3390/ani14192840 (PMC11475606; doi:10.3390/ani14192840)
Supplement: Supplementary file 1 [file animals-14-02840-s001.zip › animals-3224388- supplementary - aggie updated/Software Source Code.pdf]

```
library(sp)
library(sf)
library(devtools)
library(raster)
library(calibrate)
library(randomForest)
library(geosphere)
library(Distance)
################################################################################
Kiang <- read.csv("Data of kiang.csv", header = T)
data <- Kiang
mean(Kiang$size);sd(Kiang$size)
sum(Kiang$size[Kiang$distance<=1000])
length(Kiang$size[Kiang$distance<=1000])
ds.fit <- ds(Kiang,    convert_units = 0.001)
set.seed(1)
################################################################################
distanceSampling = function(survey){
  KEY = c("hn", "unif", "hr")
  ADJ = c("cos", "herm", "poly")
  AICs = data.frame(ID=1:9, Key = NA, Adjustment = NA, AIC = NA)
  N = 0
  for (i in 1:length(KEY)){
    for (j in 1:length(ADJ)){
      N = N+1
      AICs$Key[N] = KEY[i]
      AICs$Adjustment[N] = ADJ[j]
      ds.fit <- ds(survey, key = KEY[i], adjustment = ADJ[j], convert_units = 0.001)
      AICs$AIC[N] = as.numeric(AIC(ds.fit)[2])
```

```
            print(paste('Finished ', round(N/9*100, 2), "%", sep=''))
        }
    }
    AICs = AICs[order(AICs$AIC), ]
    return(AICs)
}
######################################################################################
AICs = distanceSampling(Kiang[Kiang$distance<=1000,])
AICs = AICs[!is.na(AICs$AIC),]
######################################################################################
ds.Kiang <- ds(Kiang, key = AICs$Key[1], adjustment = AICs$Adjustment[1], truncation=1000)
summary(ds.Kiang)
SM = summary(ds.Kiang)
Average.p = SM$ds$average.p; Average.p
error.survey = 1 - Average.p # survey uncertainty
par(mfrow=c(1,1))
######################################################################################
xlab_text <- " Distance"
ylab_text <- " Detection Probability"
plot(ds.Kiang,
          main = paste("Key:", " Hazard-rate", "\n", " Adjustment:", " cosine", sep = " "),
          xlab = xlab_text,
          ylab = ylab_text)
######################################################################################
N_estimated = 467.8155640
SE_N_estimated = 127.99475180
total_area_km2 = 883.253458
######################################################################################
density_per_km2 = N_estimated / total_area_km^2
```

CI_density_per_km2=SE_N_estimated / total_area_km^2

95%

Z_95 = 1.96

CI_lower = N_estimated - Z_95 * SE_N_estimated

CI_uKianger = N_estimated + Z_95 * SE_N_estimated

CI

CI_lower_density = CI_lower / total_area_km2

CI_uKianger_density = CI_uKianger / total_area_km2

###
